# Applicability and Limitations in the Characterization of Poly-Dispersed Engineered Nanomaterials in Cell Media by Dynamic Light Scattering (DLS)

**DOI:** 10.3390/ma12233833

**Published:** 2019-11-21

**Authors:** Arianna Marucco, Elisabetta Aldieri, Riccardo Leinardi, Enrico Bergamaschi, Chiara Riganti, Ivana Fenoglio

**Affiliations:** 1 Department of Chemistry, University of Torino, 10125 Torino, Italy; ariannamaria.marucco@unito.it (A.M.); riccardo.leinardi@unito.it (R.L.); 2 Department of Public Health and Pediatrics, University of Torino, 10126 Torino, Italy; enrico.bergamaschi@unito.it; 3 Department of Oncology, University of Torino, 10126 Torino, Italy; elisabetta.aldieri@unito.it (E.A.); chiara.riganti@unito.it (C.R.)

**Keywords:** nanomaterials, dispersion, hydrodynamic diameter, standardization, cytotoxicity

## Abstract

The dispersion protocol used to administer nanomaterials (NMs) in in vitro cellular tests might affect their toxicity. For this reason, several dispersion procedures have been proposed to harmonize the toxicological methods, allowing for the comparison of the data that were obtained by different laboratories. At the same time, several techniques and methods are available to monitor the identity of the NMs in the cell media. However, while the characterization of suspensions of engineered NMs having narrow size distribution may be easily performed, the description of aggregated NMs forming polydispersions is still challenging. In the present study, sub-micrometric/nanometric TiO_2_, SiO_2_, and CeO_2_ were dispersed in cell media by using two different dispersion protocols, with and without albumin (0.5%) and with different sonication procedures. Dynamic Light Scattering (DLS) was used to characterize NMs in stock solutions and culture media. Pitfalls that affect DLS measurements were identified and, guidance on a critical analysis of the results provided. The NMs were then tested for their cytotoxicity (LDH leakage) toward murine macrophages (RAW 264.7) and PMA-activated human monocytes (THP-1). As markers of pro-inflammatory response, nitric oxide (NO) and cytokine IL-1β production were measured on RAW 264.7 and THP-1 cells, respectively. The pre-treatment with albumin added to a strong sonication treatment increases the stability and homogeneity of the suspensions of nanometric samples, but not of the submicrometric-samples. Nevertheless, while TiO_2_ and CeO_2_ were non-cytotoxic in any conditions, differences in cytotoxicity, NO, and IL-1β releases were found for the SiO_2_, depending upon the protocol. Overall, the results suggest that there is no one-fits-all method valid for all NMs, since each class of NMs respond differently. The definition of validated procedures and parameters for the selection of the most appropriate method of dispersion for each class of NM appears to be a more efficacious strategy for the harmonization of the dispersion protocols.

## 1. Introduction

There is a growing perception regarding the importance of standardization of protocols used for the in vitro assessment of the hazard related to nanomaterials (NMs) and nano-biomaterials (NBMs) [1]. Many different models and protocols have been proposed by different Research Projects, and they are currently used and developed in the different laboratories, making the extrapolation of toxicological data to be used for regulatory purposes sometimes unsuitable. One of the main recognized sources of variability in the data that were obtained in toxicological tests among different laboratories is the different protocols used to deliver NMs to target cells in vitro [2]. 

Unlike soluble substances, NMs form in aqueous media heterogeneous systems (dispersions or colloids). Dispersions are often poorly stable, since particles tend to agglomerate and, possibly, to separate from the dispersant by sedimentation. Agglomeration degree and kinetics of agglomeration are controlled by size, shape, density, and surface chemistry of the particles and by the composition of the medium. The description and the modelling of the properties of a dispersion is particularly challenging for polydispersed NMs. 

Several studies have shown that the characteristics of the suspension modulate the in vivo and in vitro response of cells to the NMs [3,4,5,6,7]. This is due to two different reasons: the first one is related to the effect of the particle size and surface chemistry on the uptake, intra-cellular localisation, and biological effects [8,9]. The second reason is related to the dose delivered to cells, which, in the case of NMs, is often only a time-dependent fraction of the administered dose. While for soluble substances the diffusion is the only process that governs the transport of the molecular/ionic species through the cell media, in the case of NMs transport is the combination of diffusion and sedimentation [10]. These two processes both depend, in different ways, by the size, shape, and effective density of the particles, and by the viscosity and density of the dispersant [11]. 

Single laboratories [12], by International Organizations, or within research projects [2] have proposed different standardized protocols of dispersion for NMs. However, it is still unclear whether these procedures are widely applicable to all NMs or only to some of them, owing to the numerous parameters involved. 

The protocol that has been developed by the NANOGENOTOX EU project [13] further modified within the NANoREG EU project [14] was selected here as standardized protocol, since it is already used in different laboratories. It was compared with a “traditional” protocol, which is commonly used for soluble substances. 

As case studies, five samples of TiO_2_, SiO_2_, and CeO_2_ NMs from the Joint Research Centre (JRC) Repository were tested toward two macrophages cell lines. These NMs have been used as benchmark materials in several projects and they have been fully characterized and tested on several models in different laboratories. 

The distribution of aggregates/agglomerates hydrodynamic diameters (d_H_) was evaluated in both stock solutions and two different cell media (RPMI and DMEM) supplemented by 10% Foetal Bovine Serum (FBS) by Dynamic Light Scattering (DLS). This technique, albeit affected by several limitations, is the most used for the characterization of colloids, and it is widely available in most toxicological laboratories. 

The samples that were prepared following the two different protocols were compared for their cytotoxicity, evaluated as LDH leakage, toward murine macrophages (RAW 264.7) and Phorbol-12-Myristate-13-Acetate (PMA) activated human monocytes (THP-1). To evaluate the activation at sub-cytotoxic doses of RAW 264.7 cells, the NO release was measured, while the activation of THP-1 cells was evaluated by measuring the pro-inflammatory cytokine IL-1β production. 

## 2. Materials and Methods

### 2.1. Materials

JRCNM02000a (alias NM-200), JRCNM01001a (alias NM-102), JRCNM02102a (alias NM-212) were obtained by the European Commission—JRC IHCP, while NM-203 and NM-100 were obtained by the Fraunhofer Institute for Molecular Biology and Applied Ecology, Germany. Table 1 reports a summary of the main properties of the nanomaterials [15,16,17]. 

Ultrapure water was obtained from a Milli Q Plus system (Millipore, Bedford, MA, USA) and it was always used freshly prepared. All of the chemicals and solvents used were at least of analytical grade. When not otherwise specified, reagents were purchased from Sigma–Aldrich (Steinheim, Germany).

### 2.2. Thermogravimetric Analysis

Fourier transformed infrared spectroscopy (TGA-FTIR): weight loss of SiO_2_ samples analysis and investigation of the species that were released from the heated particles was carried out through the TGA-FTIR technique (Perkin-Elmer, Waltham, MA, USA). The measurement was operated in a flow of N_2_ (35 cm^3^/min.), at a heating rate of 15 °C/min., from 35 °C to 600 °C. 1–3 mg of the sample was heated in each run. The FTIR analysis of the gas evolved was carried out with a Spectrum 100 (Perkin-Elmer) spectrometer, over a wavenumber region of 600–4000 cm^−1^. The peaks of IR absorbance of both samples were recorded.

### 2.3. Preparation of the Suspensions: Standardized Protocol

Standardized protocol of dispersion the Nanogenotox protocol [13], as implemented by a standardized calibration of the sonication procedure within the NANoREG project [14], was selected. Briefly, a stock solution was firstly prepared by adding 15.36 mg of the powders to a 10 mL tube. A pre-wetting procedure was performed by adding 30 µl ethanol. Finally, 970 μL of 0.05% bovine serum albumin (BSA) in water was firstly added to the powderl followed by the remaining 5 mL 0.05% BSA by a pipette. The suspensions were transferred in a 10 mL borosilicate glass beaker, cooled in ice, and then sonicated with a probe sonicator (Sonoplus HD3100 Bandelin, Microtip MS73, diameter 3 mm, power 100 W, amplitude 40%) for 35 min. This procedure allows for the delivery of 1.1kJ/cm^3^ of acoustic energy calculated as described in the deliverable 2.06 of the NanoReg project [14]. Please refer to ref. [13] and [14] for a detailed description of the procedure. 

### 2.4. Preparation of the Suspensions: Traditional Protocol

In the traditional protocol, 15.36 mg of the powders was suspended in 6 mL of ultrapure water in a 10 mL borosilicate glass beaker. The suspension was first stirred by a Vortex™ and then sonicated with a probe sonicator (Sonoplus HD3100 Bandelin, Microtip MS73, diameter 3 mm, power 100 W, and amplitude 40%) for 5 min. in ice. 

NMs suspensions were directly diluted in the cell media. Titania and ceria samples were delivered to cell culture under reduced illumination to avoid any photo-activation of the NM. 

### 2.5. Dynamic Light Scattering Analysis

The hydrodynamic diameters distribution of the NMs suspensions that were prepared as described in the previous section was evaluated by using a Zetasizer instrument (Zetasizer Nano-ZS, Malvern Instruments, Worcestershire, UK) based on the dynamic light scattering (DLS) technique. The analyses were performed just after sonication for the stock suspensions, and after 0 or 48 h in the cell media. 

Instrument setting: replicates 10, delay time 0, equilibrium time 5 min., T = 25 °C, Dispersant refractive index, and viscosity in stock solution: 1.330/0.8872 mPa s (water); Dispersant refractive index and viscosity in cell media: 1.330/0.8882 mPa (PBS); Material refractive index and absorption: 1.544/0.200 (amorphous SiO_2_); 2.490/0.100 (TiO_2_). Reliability of the measurements was controlled by using the automatic attenuator (kept between 7 and 9) and the intercept autocorrelation function (<0.9) as quality criteria [18], according to the recommendations given in the EUNCL-PCC-001 method. Count rate was also checked to monitor particle sedimentation. Three independent replicates were performed for each condition. 

The results were expressed as hydrodynamic diameters distribution in intensity (average of mean values of 10 measurements that were obtained in three independent experiments, i.e., 30 total), mean hydrodynamic diameter (Z-average diameter—Z_D_—or mean diameters of the main peak—d_H_), and polydispersity index (PDI) ± standard deviation. 

### 2.6. Electrophoretic Light Scattering Analysis

The Electrophoretic Light Scattering (ELS) technique (Zetasizer Nano-ZS, Malvern Instruments, Worcestershire, UK) was used to determine the ζ-potential of nanoparticles suspensions in media prepared as described in the previous section. 

### 2.7. High Resolution Transmission Electron Microscopy

Micrographs were achieved with a 3010 Jeol instrument operating at 300 kV. The NMs that were pre-incubated in the stock suspensions were dropped on a copper grid that was covered with a lacey carbon film and the solvent evaporated in air.

### 2.8. Cells

RAW 264.7 murine macrophages were kindly supplied by Prof. Diana Boraschi (Institute of Protein Biochemistry—IBP-CNR). The cells were cultured in Petri dishes in DMEM (Invitrogen Life Technologies, Carlsbad, CA, USA) supplemented with 10% foetal bovine serum (FBS) and 1% penicillin-streptomycin, and then incubated in the same culture medium for 24–48 h, in the absence or presence of the samples before the assays. The protein content of cell monolayers was assessed with the bicinchoninic acid assay.

THP-1 human monocytic cells were kindly supplied by Carolina Aristimuño (GAIKER-IK4 Centro Tecnológico, Spain). The cells were pre-treated (24 h) with 0.5 μM PMA to induce their differentiation into macrophages. Cells were cultured in Petri dishes in RPMI (Invitrogen Life Technologies, Carlsbad, CA, USA) supplemented with 10% FBS and 1% penicillin-streptomycin, and then incubated in the same culture medium for 24–48 h, in the absence or presence of the samples before the assays. The protein content of cell monolayers was assessed with the bicinchoninic acid assay.

### 2.9. Measurement of Lactate Dehydrogenase Leakage

The cytotoxic effect of the NMs was measured as lactate dehydrogenase (LDH) leakage into the extracellular medium, while using a Synergy HT microplate reader (Bio-Tek Instruments, Winooski, VT, USA), as previously described [19]. Intracellular and extracellular LDH were both measured and then extracellular LDH (LDH out) was calculated as a percentage of the total (intracellular + extracellular) LDH (LDH tot). Purified LDH (from bovine muscle, purity > 99.5%) was used to rule out the interference of NMs on the enzyme activity. Briefly, the cell culture medium in the absence (control) or presence of NMs (10–320 µg/mL) was incubated with 0.3 U/mL of LDH at 37 °C. After 24–48 h, the medium was centrifuged and enzyme activity was spectrophotometrically measured in the supernatants. 

### 2.10. Measurement of Nitrite

RAW 264.7 cells were cultured for 24–48 h in the absence or presence of the samples. The amount of extracellular nitrite (the stable derivative of nitric oxide) was spectrophotometrically measured, by adding 0.15 mL of cell culture medium to 0.15 mL of Griess reagent in a 96-well plate. After 10 min. of incubation at 37 °C in the dark, the absorbance was detected at 540 nm with a Synergy HT microplate reader. For each experiment, a blank was prepared in the absence of cells, and its absorbance was subtracted from that measured in the presence of cells. Nitrite concentration was expressed as nmoles nitrite/mg cell proteins. Bacterial LPS (lipopolisaccarhyde, 20 ug/mL) was used as the positive control (data not shown).

### 2.11. Measurement of Interleukin-1β (IL-1 β) Production

After a 24–48 h incubation of THP-1 and RAW 264.7 cells in the absence or presence of 10–25 µg/cm^2^ of NM-200 and NM-203 (prepared with traditional or standardized protocols), the extracellular medium was collected and centrifuged at 13,000 *g* for 30 min. The concentration of the cytokine was determined in the supernatant by using the conventional ELISA kit from R&D System’s (Minneapolis, MN, USA), following the instructions of the manufacturer. The absorbance was measured at 450 nm with a Synergy HT microplate reader. The cytokine amount was expressed as a percentage increase of IL-1 β versus the respective control incubated without nanomaterials (assumed as 100%).

### 2.12. Statistical Analysis

All the data are provided as means ± SEM of the data obtained by three independent experiments performed by two different operators. The results were analyzed by a one-way analysis of variance (ANOVA) and Tukey’s test.

## 3. Results

A summary of the main properties of the NMs used is in Table 1 [15,16,17]. NM-200 and NM-203 are two amorphous silica samples having similar primary particle size and specific surface area (SSA), but are synthetized by precipitation or pyrolysis, respectively. These different synthetic methods make the two silica samples different for their surface chemistry. Pyrogenic silica, in fact, exhibit a lower degree of hydrophilicity as compared with the precipitated one due to a low abundance of hydrophilic surface hydroxyl groups with respect to silica produced by wet methods [20].

TGA analysis coupled with a FTIR detector under nitrogen atmosphere was performed to confirm that the batches used here did not undergo any variation of the surface chemistry due to storage or manipulation. The percentage weight losses and the derivative curves of the two samples are compared in Figure S1 (SI). NM-200 exhibited an overall weight loss of about 5%, 2% at temperature lower than 100 °C, due to the desorption of adsorbed water, and a remaining 3% at higher temperatures ascribable to the release of water following the condensation of close hydroxyl groups to Si–O–Si bridges [21]. In fact, the analysis of the FTIR spectra showed that only water was released during heating, while no signal due to aliphatic or aromatic groups was detected, thus confirming the purity of the samples. As expected, no significant weight loss was detected for NM-203. This was expected, since the lower abundance of hydroxyl groups makes this sample less inclined to adsorb water and condense by forming siloxanes. 

NM-100 and NM-101 are titanium dioxide nanomaterials, the first one being composed by nanometric/submicrometric particles, the second one by aggregates of nanometric particles. Finally, NM-212 is a sample of cerium dioxide, composed, similarly to NM-100, by particles covering a wide interval of sizes. Both of the samples contain sub-micrometric particles [15,16,17], in agreement with the low specific surface area. 

**Figure 1:**
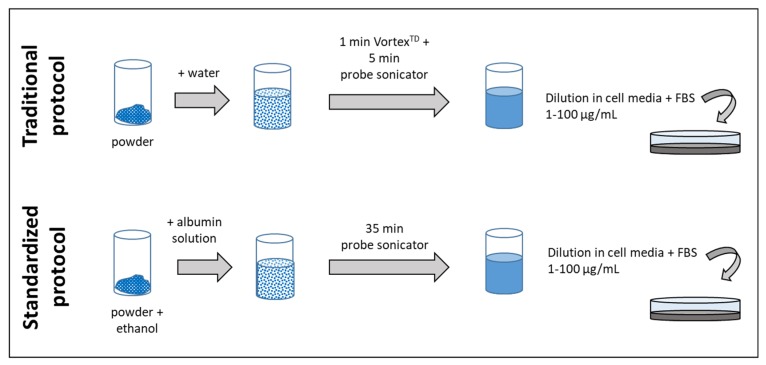
Main differences between the two protocols used to prepare the NM suspensions for in vitro tests.

The samples were prepared for toxicological tests according to two different protocols, as described in detail the method section. Figure 1 summarizes the main steps of the two protocols.

The two protocols differ for the kind of solution used to prepare the stock solution and the sonication procedure. In the traditional protocol, the NMs were suspended in ultrapure water, and mildly de-agglomerated by vortexing and probe sonication. In the standardized protocol, a small amount of ethanol was introduced to pre-wet the powder, and the powder was then suspended in water with 0.05% *w*/*v* albumin, which can generally improve dispersion stability through steric, polymeric, or electrostatic stabilization [22], and further sonicated for a time sufficient to deliver a fixed amount of energy [13,14]. 

### 3.1. Characterization of the Stock Suspensions

**Figure 2:**
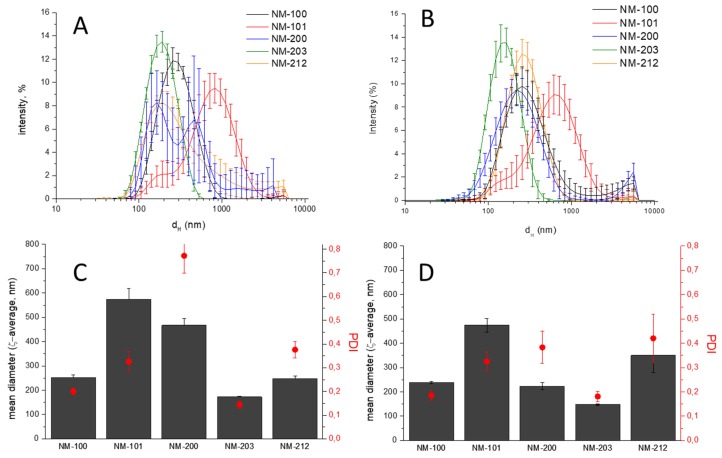
Dynamic Light Scattering (DLS) analysis of stock suspensions. (**A**,**B**) d_H_ distribution; (**C**,**D**) mean hydrodynamic diameters (Z_D_) and polydispersity index (PDI) values; traditional protocol (**A**,**C**); and, standardized protocol (**B**,**D**).

DLS measurements were immediately conducted after sonication of each of the two different batch dispersions. Figure 2 shows the intensity-based hydrodynamic size-distributions, the average zeta-size, and the polydispersivity index. The numerical values are reported in the supplementary material. 

All of the samples are characterized by particles having d_H_ distributed in a wide interval in both media. This was expected, since the powders used are composed by aggregates, and are prone to further agglomerate. Note that the intensity-based hydrodynamic size-distributions overestimate the presence of larger particles [15,16,17]. This might be avoided by converting them in number-based size distribution, as shown in the supplementary material (Figure S2). However, the algorithm that was used by the instrument for the conversion assumes diameters equivalent to spherical particles having homogenous density. 

All of the suspensions prepared according the standardized protocol have a DLS pattern similar to those prepared according the traditional protocol, with the exception of NM-200 that appears to be more stable and homogeneously distributed. A clear reduction of the Z_D_ and PDI was observed for NM-200 only by using the standardized protocol (Figure 2 and Table S1). Note that the d_H_ distribution in the stock suspension by using the standardized protocol are in line with those that were previously reported for the two silica samples [15]. 

No evident sedimentation was observed in the vials for the time needed for the DLS measurements (15–20 min.) (Figure S3). The absence of sedimentation was further confirmed by the values of the count rate, which remained nearly constant during the measurements (data not shown). However, after 1 h, a clear sedimentation was visually observed for NM-100, NM-101, and NM-212 (SM) in the suspensions prepared according the traditional protocol. After 2 h, sedimentation was observed for all samples. 

**Figure 3:**
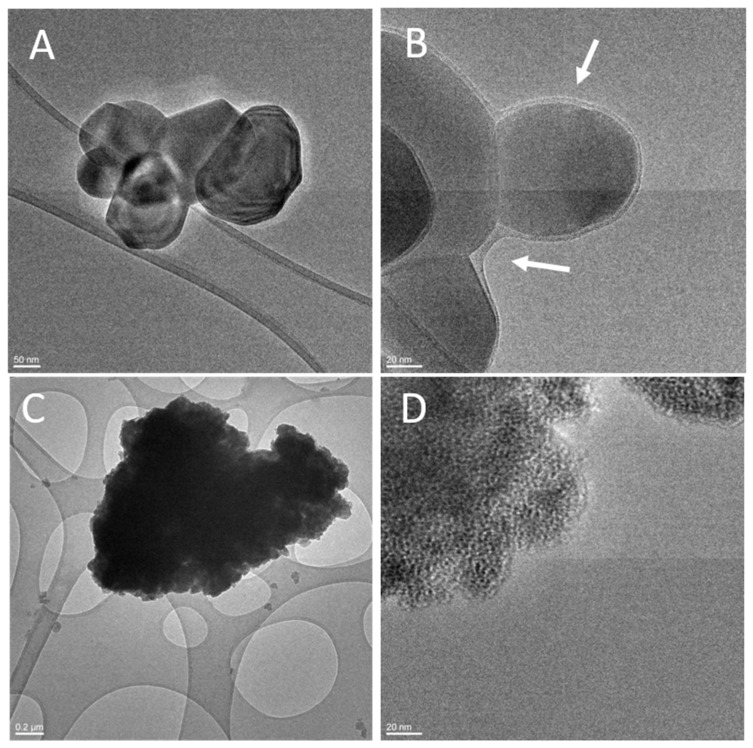
TEM analysis of the TiO_2_ NMs after incubation in the stock suspensions: (**A**,**C**) traditional protocol; (**B**,**D**) standardized protocol; (**A**,**B**) NM-100; (**C**,**D**) NM-101.

The differences observed for NM-200 prepared following the two protocols were expected, since albumin has a strong tendency to adsorb onto the surface of silica and metal oxides [23,24]. This generally makes the de-agglomeration of the sample by sonication easier, as reported in previous studies [25,26]. For the other samples, the effect of albumin was less evident, which suggests that the samples were composed in prevalence by aggregates. On the other hand, HRTEM analysis performed on the titania samples incubated with the two stock suspensions and dried without washing (Figure 3) showed an organic layer surrounding the particles, which was particularly visible in NM-100 preparation, likely due to albumin. The layer might be a consequence of the presence of a protein corona since no aggregates of organic matter due to the mere elimination of the solvent were observed in the sample.

### 3.2. Characterization of NMs Cell Media: Effect of the Concentration

**Figure 4:**
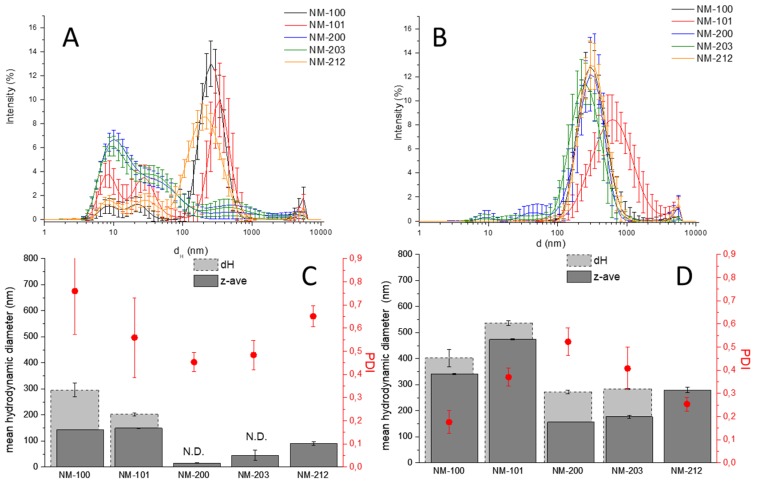
DLS analysis of the NM in RPMI. (**A**,**B**) d_H_ distribution; (**C**,**D**) mean hydrodynamic diameters (Z_D_ dark grey bars and d_H_ light grey bars) and PDI values; nanomaterials concentration: 1 (**A**,**C**) and 100 μg/mL (**B**,**D**) N.D.: not determined.

DLS analysis of the NMs in the media supplemented by FBS was firstly performed at the lowest (1 µg/mL) and highest (100 µg/mL) concentration of the NMs used in cellular experiments. The DLS patterns that were obtained by the five NMs in RPMI, the Z_D_ values and the PDI are reported in Figure 4. Each curve represents the mean values of 30 measurements that were obtained in three independent experiments. Therefore, the extent of the bars, representing the standard deviation, gives information regarding the stability of the suspensions during the time of each measurement (15–30 min.) and the repeatability of the three measures in the three independent experiments. Note that the presence of aggregates/agglomerates having an equivalent hydrodynamic diameter higher than 1 μm cannot be detected by DLS. Moreover, the intensity of the peaks does not reflect the relative abundance of the various population.

Lower Z_D_ values (dark grey bars) were observed for all samples in the diluted suspension with respect to the concentrated ones, an effect particularly obvious in the case of silica. However, looking at the DLS patterns of the diluted suspensions, two broad populations having diameters in the range of 3–100 nm were observed in all suspensions. These populations are not due to the NMs, but to the proteins of the serum, as confirmed by the DLS analysis of the media (SI, Figure S4). The mean d_H_ values of the most intense peak are also reported in Figure 4C,D (light grey bars). The comparison of d_H_ with the Z_D_ shows that the second index underestimates the mean hydrodynamic diameter value, with the largest effect in diluted suspensions, due to the presence of the proteins.

Such an effect is particularly evident for silica preparations: this nanomaterial has, in fact, a refractive index near to that of water, making the scattered light intensity very low. The present results suggest the caution of Z_D_ as parameter to describe agglomeration state of NMs in media containing proteins, since it underestimates the mean size of agglomerated particles in a poor predictable way. The mean hydrodynamic diameter of each single populations appears to be a better index for monodispersed or low medium polydispersed NMs. On the other hand, the identification of the populations for samples having a low scattering intensity might be unfeasible, like for silica where the peak correspondent to the NM was absent. Similar results were obtained on DMEM medium (data not shown).

### 3.3. Characterization of NM in Cell Media: Stability During the Time of Incubation

**Figure 5:**
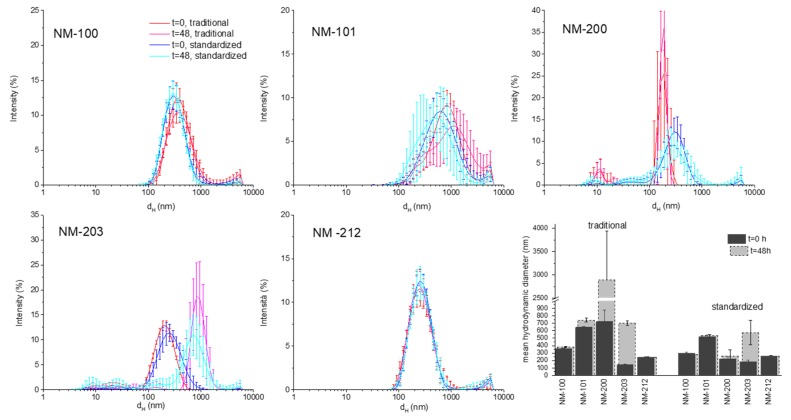
DLS analysis of the NM in RPMI at 100 µg/mL at t = 0 and t = 48 h. The panels represent for each sample the comparison between the suspensions prepared by the traditional protocol and those prepared by the standardized protocol. In the last panel, the mean Z_D_ ± SD are reported. Data are the mean of 30 measurements.

**Figure 6:**
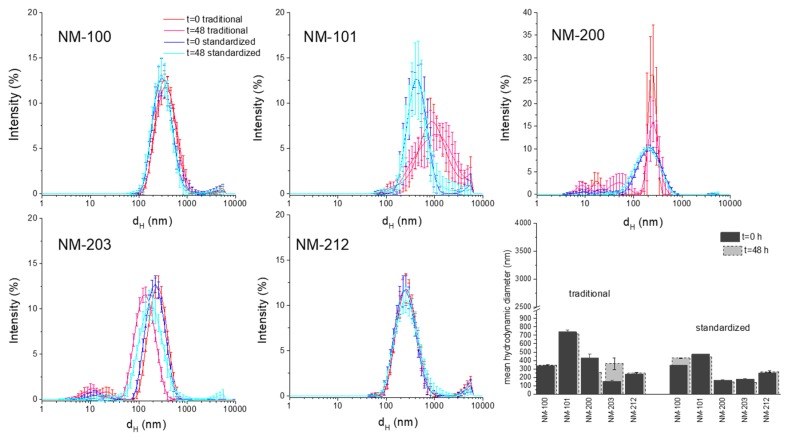
DLS analysis of the NM in DMEM at 100 µg/mL at t = 0 and t = 48 h. The panels represent for each sample the comparison between the suspensions prepared by the traditional protocol and those prepared by the standardized protocol. In the last panel, the mean Z_D_ ± SD are reported. Data are the mean of 30 measurements.

The behaviour of the five NMs in cell media (RPMI and DMEM) was evaluated by DLS analysis just after dilution and after 48 h of incubation at the highest concentration (100 µg/mL). The composition of the cell media, as declared by the provider, is reported in Table S2. In Figure 5 and Figure 6, the comparison between the DLS patterns of the suspensions prepared by the traditional protocol and those that were prepared by the standardized protocol at two time points (0 and 48 h) is reported. A summary of the Z_D_ values is also reported in the latest panel of the figures. ζ-potential values are reported in the SM. 

In general, all of the samples show a similar dispersion in the cell media as compared to the dispersions in the stock suspensions. This was expected, due to the nature of the samples and the low electrostatic repulsion between particles, as inferred by the low ζ-potential values measured (Table S3). However, the different behaviour was observed, depending upon the material. The colloid formed by the sub-micrometric titanium dioxide (NM-100) showed high stability. The overlapping of the peaks and the similar count rate (data not shown) suggested that NM did not agglomerate or sediment during incubation time in both media and with both protocols. Moreover, the d_H_ distribution was similar to those in the stock solutions. A similar behaviour was observed for the cerium dioxide sample (NM-212), which appeared to form stable and low dispersed suspension in both media. 

The nanometric materials, more prone to agglomeration, showed a different behaviour. NM-101 formed in RPMI and DMEM suspensions that are very unstable, as inferred by the variability of the distribution pattern and by the high PDI value (data not reported). The suspension that was prepared with the standardized protocol in DMEM appeared to be more uniform in size distribution, more stable, and with lower Z_D_ as compared to those that were prepared with the traditional protocol. This effect was not observed in RPMI. This was unexpected, since composition, pH, and osmolarity of the two media, are very similar (SI). An evident stabilizing effect of the standardized protocol was observed in the case of the silica sample NM-200. The DLS pattern of the suspension that was prepared with the traditional protocol exhibits a large variability. Moreover, the appearance at 48 h of a peak with a maximum at 10 nm correspondent to proteins suggested a partial sedimentation due to the formation of agglomerates. When prepared with the standardized protocol, the dispersions of NM-200 appeared to be less heterogeneous and more stable during time. NM-203 appeared to be more uniformly distributed during time than NM-200 in all conditions. However, a clear agglomeration occurred during time in RPMI, but not in DMEM. The kind of dispersion protocol had little effect on this samples in both media.

### 3.4. Cytotoxicity Toward Macrophages

NMs cytotoxicity was assessed by measuring the leakage of the intracellular enzyme lactate dehydrogenase (LDH). LDH leakage is a consequence of cellular membrane damage following a direct mechanical perturbation of the membrane by the particles or as a consequence of peroxidation, which, in turn, is an indication of oxidative stress. 

The titanium dioxide and cerium dioxide NMs did not induce any cytotoxic effect at any of the dose tested in both cell lines (Figures S5 and S6), in agreement with data previously published [27]. For these samples, the different preparation protocols have no effect on cytotoxicity. Conversely, in the case of silica NMs, a mild cytotoxic effect was observed at higher doses. 

**Figure 7:**
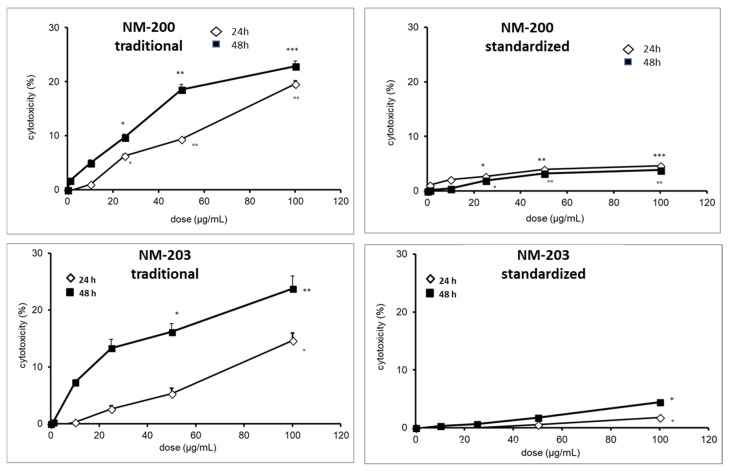
Effect of the dispersion protocols on the cytotoxicity of NM-200 and NM-203 towards PMA-activated human monocytes (THP-1) cells measured as lactate dehydrogenase (LDH) leakage. Each measurement was performed in duplicate, and data are presented as means ± SEMs (n = 3 readings): *** *p* < 0.0001 vs. ctrl; ** *p* < 0.001 vs. ctrl; * *p* < 0.01 vs. ctrl; °° *p* < 0.001 vs. ctrl; ° *p* < 0.01 vs. ctrl.

**Figure 8:**
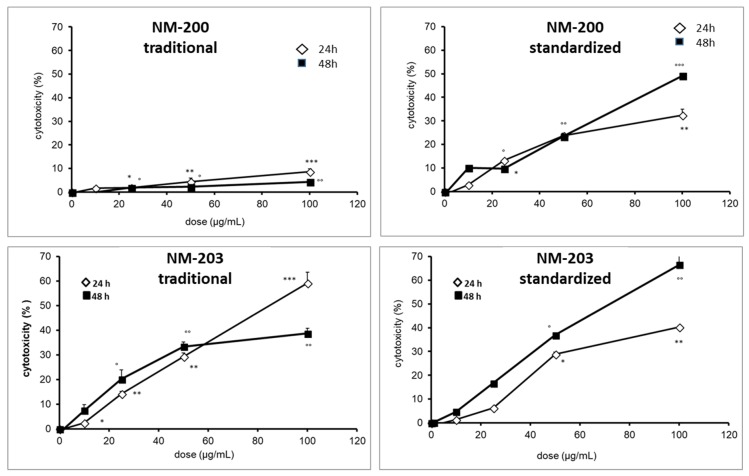
Effect of the dispersion protocols on the cytotoxicity of NM-200 and NM-203 towards RAW 264.7 cells measured as LDH leakage. Each measurement was performed in duplicate, and data are presented as means ± SEMs (n = 3 readings): *** *p* < 0.001 vs. ctrl; ** *p* < 0.002 vs. ctrl; * *p* < 0.05 vs. ctrl; °° *p* < 0.001 vs. ctrl; ° *p* < 0.02 vs. ctrl.

In Figure 7 and Figure 8, the cytotoxicity of the two silica samples that were prepared by the two different protocols toward THP-1 (RPMI media) and RAW 264.7 (DMEM media), respectively, are reported.

Different effects were observed on both of the macrophage lineages, depending upon the dispersion protocol. In THP-1 cells, when the traditional protocol was used, the two silica samples elicited a cytotoxic effect at 24 h lower than those that were observed at 48 h, whereas applying the standardized protocol the effect at 24 h was close to that observed at 48 h. This is consistent for NM-200 with the higher instability being observed in the suspensions prepared according the traditional protocols (Figure 6), which suggests that sedimentation might have an additional contribution to the transport of the particles close to the cells. Conversely, the differences in cytotoxicity that were observed for NM-203 did not find any correspondence with the measurement performed with DLS, suggesting the contribution of factors other than size. Notably, when the standardized protocol was used on THP-1 cells, a clear suppression of the cytotoxicity was observed for both of the samples. 

The samples also elicited different effects on the RAW 264.7 cell line (Figure 8). As in THP-1 cells, NM-203 was more cytotoxic than NM-200. However, the effect was more pronounced when the samples were prepared following the traditional protocol. Conversely to THP-1, no inhibitory effect of cytotoxicity in the standardized protocol was observed for RAW 264.7 cells.

Note that no activation was observed for NM-100, NM-101 and NM-212 (Figure S7).

### 3.5. Activation of RAW 264.7 and THP-1 Cells

**Figure 9:**
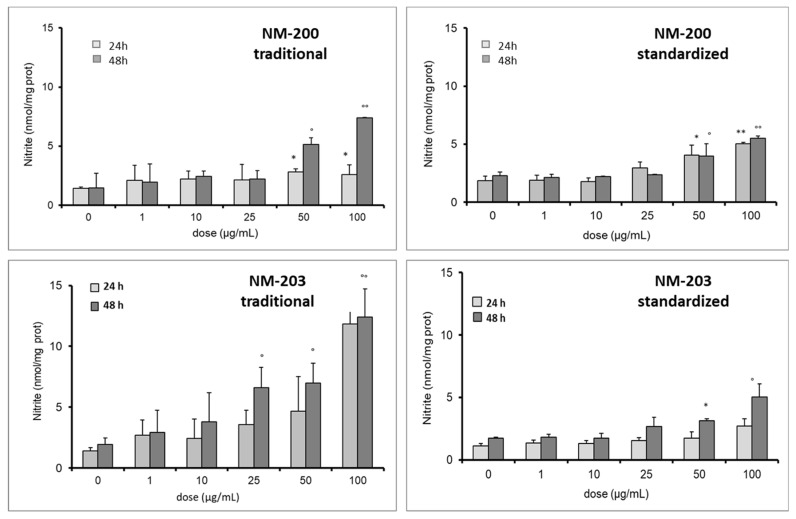
Effect of the dispersion protocols on the induction of NO release by RAW 264.7 cells by NM-200 and NM-203. Each measurement was performed in duplicate, and data are presented as means ± SEMs (n = 3 readings): ** *p* < 0.01 vs. ctrl; * *p* < 0.05 vs. ctrl; °° *p* < 0.001 vs. ctrl; ° *p* < 0.05 vs. ctrl.

The ability of the two materials to induce a pro-inflammatory activation on RAW 264.7 cells was evaluated by measuring the release of NO in the extracellular medium (Figure 9). 

The pro-inflammatory activation was evaluated in these cells as the release of the cytokine IL-1β since the NO production by THP-1 cells is negligible, as previously reported [28]. 

Both silica samples induced a significant production of NO by macrophages in RAW 264.7 cells at the higher doses. However, the effect was lower when NMs were prepared with standardized protocol at 24 and 48 h similarly to that observed for cytotoxicity. Moreover, when the traditional protocol was used, a large variability of the data was observed, in agreement with the DLS data. 

**Figure 10:**
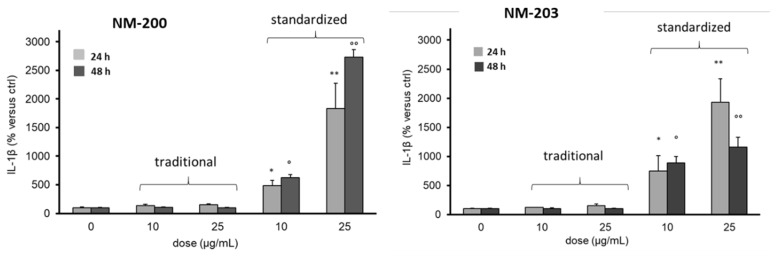
Effect of the dispersion protocols on extracellular levels of Interleukin-1β (IL-1 β) in THP-1 cell cultures by NM-200 and NM-203. Cells were incubated for 24–48 h in the absence (0, control) or presence of NM-200 and NM-203 at a concentration of 10 and 25 μg/mL. The results are expressed as percentage increase of IL-1 β vs. the respective control incubated without NM-200 or NM-203 (assumed as 100%). Each measurement was performed in duplicate, and data are presented as means ± SEMs (n = 3 readings): * *p* < 0.01; ** *p* < 0.0001; ° *p* < 0.005 °° *p* < 0.0001.

The activation of THP-1 cells at sub cytotoxic doses (10 and 25 µg/mL) was measured as a release of cytokine IL-1β (Figure 10). 

## 4. Discussion

One of the most debated issues in the assessment of NMs hazard is the poor consistency among the data generated by different laboratories. To address this issue, several studies focusing on the assessment of the delivered dose and on the characterization of the NMs in the culture media have been published [6,11,26,29]. Another debated aspect, which affects the predictive power of the toxicological tests, is at which extent they reproduce realistic conditions [30]. Both issues are relevant for both NBMs and for NMs to whom humans may be accidentally exposed. However, while NBMs have controlled properties, NMs in the environment or in occupational settings exhibit a variability in their physico-chemical properties that depends upon the intrinsic properties of the material, but also by the context. This makes the development of appropriated methods for hazard assessment reflecting real life situations challenging. For instance, NBMs designed for systemic delivery need to be monodispersed and stable in the suspensions used in the toxicological tests, similarly to what expected in vivo. Instead, to reproduce the real conditions for NMs used as filler or additives, typically strongly aggregated and with a wide distribution of size and a strong tendency to agglomerate, is more challenging. 

Nevertheless, the development of standardized protocols for toxicological tests is necessary for both industries and regulatory agencies. The protocols and the technique used need to be widely available at acceptable costs. Furthermore, the appropriateness of each proposed protocol to the different class of NMs needs to be proven due to the large variability in the properties of NMs. 

In traditional toxicological tests, soluble substances are firstly dissolved in water to generate a stock suspension, and then diluted in the media. NMs that are originally produced in the form of colloids may be processed in a similar way, while powders need a suitable de-agglomeration process. Different techniques can be used to de-agglomerate NMs, such as mechanical stirring, vortexing, bath, or probe sonication. The addition of surfactants (dispersants, natural surfactants, proteins) has been also proposed for both hydrophilic and hydrophobic NMs. The addition of molecules acting as surfactants improves the reproducibility of the tests by stabilizing the suspension. However, to what extent these substances mirrors physiological conditions is questionable. Proteins are a good alternative, since they are already added as nutrients for cells into the media and they are naturally occurring in biological fluids.

In the present study, the standardized protocol that was developed within the EU NANOGENOTOX, further implemented following the indication of the NANoREG deliverable 2.06 that use albumin as dispersant and a standardized sonication procedure, was compared with a traditional protocol, in which the NMs are simply dispersed and shortly sonicated in water. 

DLS monitored the size distribution of the samples in the suspensions. Albeit, the characterization of polydispersed samples by this technique is biased by several factors [31], this technique is commonly used to characterize colloids due to the relatively low cost of the instruments. ISO (ISO 22412:2017), EU, and US Nanotechnology Characterization Laboratories (NCL) have published methods for DLS measurements. 

In the EUNCL method EUNCL-PCC-001 (http://www.euncl.eu), several factors that may generate artefacts in the measurements of size by DLS are listed. In addition, our data underline the interference of proteins on the measurement on dilute samples, also when there is no overlapping among populations. This interference is poorly predictable, since it depends upon both concentration and kind of NM, being more important for materials having a refractive index close to water. On the other hand, the presence of this interference might be revealed by comparing the Z_D_ value with the mean d_H_ of the main peak, which must be reported, when possible. 

Information regarding the stability of the suspensions might be interfered by a critical analysis of different parameters, i.e., variation of the values of count rate, the repeatability of the measurements evaluated by the amplitude of the standard deviation bars, the overlapping of the distribution curves, and the appearance of the pick correspondent to proteins centred at 10 nm during time. 

When considering all of the above mentioned limitations, the present data clearly show that the presence of albumin and a prolonged sonication improves the stability of the stock suspensions and reduces the mean hydrodynamic diameter in cell media for the NMs having nanometric primary particles (NM-101, NM-200, and at a less extent NM-203). Conversely, for the samples that were composed by sub micrometric particles, the effect is negligible. 

While the higher stability of the suspensions that were prepared by the standardized protocol might reflect on the precision of the data obtained in the cellular tests, the reduction of the hydrodynamic diameters is expected to modify both cell response and kinetic of transport of particles in the media. At the same time, the presence of a protein corona modifies the response of cells to the NMs. 

The effect that was observed on nanometric samples might be due to both the formation of a hard protein corona that increases the repulsion among particles or to the more prolonged sonication procedure that facilitates de-agglomeration of the particles.

Albumin is largely present in the cell media, being the most abundant protein in the FBS [32,33]. Consequently, it should be abundant in the protein coronas of NMs that were prepared with both protocols. On the other hand, the protein corona composition is determined by a peer competition of the different proteins for the surface [34]. The presence of albumin pre-adsorbed might inhibit the adsorption of proteins with more affinity for the surface, thus modifying the composition of the protein corona. 

The inhibition of NM toxicity by proteins has been reported in previous studies [33,35,36]. Albumin is commonly used to improve the biocompatibility and the time of permanence in circulation of nanoparticles, but it might also facilitate the uptake of nanoparticles [37]. In other cases, proteins were found to enhance the toxicity of NMs [38]. In the case of silica, the inhibitory effect of albumin in acute toxicity was expected due to the masking of the surface by the proteins, which might reduce the surface reactivity [39]. However, we show here that, in some cases, an enhancement of the toxic effect might be observed. 

A clear correlation between the DLS and cellular data was not observed. In fact, while the protocol that was used to prepare the suspension similarly affects the size distribution of the SiO_2_ samples_,_ enhancement or reduction of the toxicity were observed. In the case of THP-1 cells, the standardized protocols mitigate the cytotoxic effect of silica, but increase the activation of THP-1 cells, in which an onset of inflammation could be induced. 

In RAW 264.7 cells, an enhancement of the cytotoxic effect was observed for NM-200 with the standardized protocol, while both of the silica samples prepared with the traditional protocol induced a release of NO higher than the samples prepared with standardized protocol. 

Moreover, it is worth of note that, while overall the pyrogenic silica sample NM-203 exhibited a higher cytotoxic effect with respect to the precipitated silica NM-200, in agreement with several previous studies [20,21,39,40] the differences among the two silica NMs were mitigated when the standardized protocol was used. 

**Figure 11:**
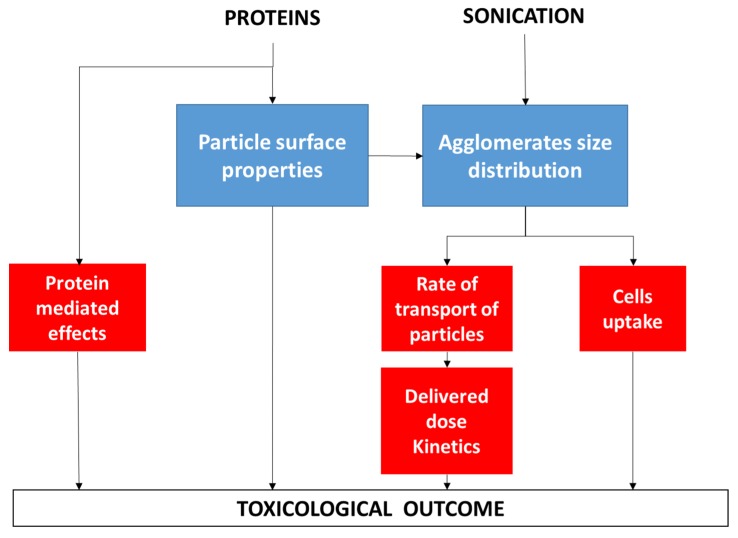
Summary of the possible effects of proteins and sonication on the nanomaterials toxicity. The formation of a protein corona affects the surface properties of the particles (charge, reactivity, topography) that in turn may affects both agglomerates size distribution and cell response. Protein mediated effects may occur depending upon the protein corona composition and the arrangement of the proteins at the surface. Sonication affects the agglomeration size distribution that in turn modulate the rate of transport and the cell uptake.

The poor consistency between the differences in size distribution that was monitored by DLS and the toxicological results might be explained by the multiple effects that proteins and sonication may have on the properties of the nanomaterials, as summarized in Figure 11, which are, in turn, dependent on the nature of the nanomaterial. 

## 5. Conclusions

The data reported herein confirm the importance to harmonize the methods for the administration of NMs and NBMs to cells in hazard assessment among laboratories. At the same time, they discourage the use of a single protocol for all NMs, suggesting, as an alternative strategy, the implementation of available validated experimental protocol and parameters, which in turn can be used to set-up appropriate dispersion procedures for defined class of NMs. In this contest, the present study contributes to the development of reliable DLS-based methods for the monitoring of polydisperse NMs in cell media for quality control purposes. 

## Figures and Tables

**Table 1:** Main physico-chemical properties of the samples [15,16,17].

Name	Composition Crystallinity ^a^	Impurities (>0.01%) ^b^	Specific Surface Area ^c^ (m^2^/g)	Primary Particles Diameter (range, nm) ^d^	Z-Average (nm) ^e^
JRCNM02000a (alias NM-200) *	precipitated amorphous SiO_2_ (96%)	Na, Al, Ca, S	189.16	10–20	≈200
**NM-203**	pyrogenic amorphous SiO_2_ (99%)	Na, Al, Ca, S	203.92	10–11	140–240
**NM-100**	TiO_2_, anatase (97.7%)	P, K	9.230	20–300	228.6
JRCNM01001a (alias NM-101) *	TiO_2_, anatase (98.1%)	Al, Na, P, S	316.07	5–6	Not reported
JRCNM02102a (alias NM-212) *	CeO_2_, cerianite (82.62%)	Al	27.2	10–100	Not reported

^a^ EDS and XRD; ^b^ ICP-OES ^c^ BET method; ^d^ TEM/SEM; ^e^ DLS in water. Note that more than one values are reported in the reference. * All samples are hereafter referred to as NM-100, NM-101, NM-200, NM-203, and NM-212.

## Supplementary Materials

The following are available online at https://www.mdpi.com/1996-1944/12/23/3833/s1, Table S1: Z-average and PDI measured on the stock suspensions of the NMs in the media following the traditional and standardized protocol; Table S2: Composition of the cell media DMEM and RPMI as declared by the provider; Table S3: ζ -potential measured on the suspensions of the NMs in the media following the traditional and standardized protocol. (NM concentration 100µg/mL); Figure S1: TGA weight loss curves for JRC-200 and NM-203, under N_2_ flow; Figure S2: d_H_ distribution (number) of stock suspensions; Figure S3: Images of the stock suspension prepared by the traditional protocol just after sonication, after 1 h and after 2 h; Figure S4: DLS analysis of the RPMI media; Figure S5: Effect of the dispersion protocols on the cytotoxicity of NM-100, 101 and 212 toward THP-1 cells measured as LDH leakage; Figure S6: Effect of the dispersion protocols on the cytotoxicity of NM-100, 101 and 212 toward RAW 264.7 cells murine macrophages measured as LDH leakage; Figure S7: Effect of the dispersion protocols on the induction of NO release by RAW 264.7 murine macrophages by NM-100, 101 and 212.
