# Supplementary material for: Applicability and Limitations in the Characterization of Poly-Dispersed Engineered Nanomaterials in Cell Media by Dynamic Light Scattering (DLS)
Source: Materials (Basel). 2019 Nov 21;12(23):3833. doi: 10.3390/ma12233833 (PMC6926523; doi:10.3390/ma12233833)
Supplement: Supplementary file 1 [file materials-12-03833-s001.pdf]

materials

MDPI

Supplementary Materials

Applicability and limitations in the characterization of poly-dispersed engineered nanomaterials in cell media by Dynamic Light Scattering (DLS).

Arianna Marucco [1,2], Elisabetta Aldieri [3], Riccardo Leinardi [1], Enrico Bergamaschi [2], Chiara Riganti [3], and Ivana Fenoglio [1,*]

[1] Department of Chemistry, University of Torino, 10125 Torino, Italy; ariannamaria.marucco@unito.it (A.M.); riccardo.leinardi@unito.it (R.L.);
[2] Department of Public Health and Pediatrics, University of Torino, 10126 Torino Italy enrico.bergamaschi@unito.it
[3] Department of Oncology, University of Torino, 10126 Torino, Italy; elisabetta.aldieri@unito.it (E.A.); chiara.riganti@unito.it (C.R.)
* Correspondence: ivana.fenoglio@unito.it; Tel.: +39 6707506

Table S1. Z-average and PDI measured on the stock suspensions of the NMs in the media following the traditional and standardized protocol.

Sample	Z-Average (nm) Traditional Protocol	PDI Traditional Protocol	Z-Average (nm) Standardized Protocol	PDI Standardized Protocol
**NM-200**	467.4 ± 26.6	0.772 ± 0.074	238.4 ± 3.9	0.185 ± 0.019
**NM-203**	172.1 ± 2.3	0.144 ± 0.015	147.7 ± 3.7	0.181 ± 0.022
**NM-100**	252.8 ± 10.2	0.200 ± 0.014	222.7 ± 14.7	0.383 ± 0.066
**NM-101**	573.3 ± 44.2	0.326 ± 0.042	473.5 ± 27.7	0.325 ± 0.039
**NM-212**	248.3 ± 11.1	0.376 ± 0.035	349.8 ± 72.2	0.420 ± 0.100

Table S2. Composition of the cell media DMEM and RPMI as declared by the provider.

	**DMEM**	**RPMI**
**Osmolality**	260–310 mOsm/kg	300–340 mOsm/kg
**pH**	7.0–7.4	7.0–7.2
**Amino Acids** (mM)		
**Glycine**	0.4	0.13333334
**L-Alanyl-Glutamine**	3.9723501	2.0552995
**L-Arginine hydrochloride**	0.39810428	1.1494253
**L-Asparagine**	-	0.37878788
**L-Aspartic acid**	-	0.15037593
**L-Cystine**	0.15335463	0.20833333
**L-Glutamic Acid**	-	0.13605443
**L-Histidine hydrochloride-H_2O**	0.2	0.09677419
**L-Hydroxyproline**	-	0.15267175
**L-Isoleucine**	0.8015267	0.3816794
**L-Leucine**	0.8015267	0.3816794
**L-Lysine hydrochloride**	0.7978142	0.21857923
**L-Methionine**	0.20134228	0.10067114
**L-Phenylalanine**	0.4	0.09090909
**L-Proline**	-	0.17391305
**L-Serine**	0.4	0.2857143
**L-Threonine**	0.79831934	0.16806723
**L-Tryptophan**	0.078431375	0.024509804
**L-Tyrosine**	0.39779004	0.110497236
**L-Valine**	0.8034188	0.17094018
**Vitamins** (mM)		
**Biotin**	-	8.1967213×10^{-4}
**Choline chloride**	0.028571429	0.021428572
**D-Calcium pantothenate**	0.008385744	5.24109×10^{-4}
**Folic Acid**	0.009070295	0.0022675737
**Niacinamide**	0.032786883	0.008196721
**Para-Aminobenzoic Acid**	-	0.00729927
**Pyridoxine hydrochloride**	0.019417476	0.004854369
**Riboflavin**	0.0010638298	5.319149×10^{-4}
**Thiamine hydrochloride**	0.011869436	0.002967359
**Vitamin B12**	-	3.690037×10^{-6}
**i-Inositol**	0.04	0.19444445
**Inorganic Salts** (mM)		
**Calcium nitrate**	-	0.42372882
**Calcium Chloride**	1.7959183	-
**Ferric Nitrate**	2.4752476E-4	-
**Magnesium Sulfate**	0.8130081	0.40650406
**Potassium Chloride**	5.3333335	5.3333335
**Sodium Bicarbonate**	44.04762	23.809525
**Sodium Chloride**	110.344826	103.44827
**Sodium Phosphate monobasic**	0.90384614	5.633803
**Other Components** (mM)		
**D-Glucose (Dextrose)**	5.5555553	11.111111
**Phenol Red**	0.039851222	0.0032573289
**Sodium Pyruvate**	1.0	0.013283741

Table S3. ζ -potential measured on the suspensions of the NMs in the media following the traditional and standardized protocol. (NM concentration 100μg/mL).

Media	Sample	ζ-Potential (mV) Traditional Protocol	ζ-Potential (mV) Standardized Protocol
DMEM	**NM-200**	−10.5 ± 0.5	−6.65 ± 1.05
	**NM-203**	−10.9 ± 1.1	−7.33 ± 5.21
	**NM-100**	−12.1 ± 0.5	−5.50 ± 1.18
	**NM-101**	−13.8 ± 1.6	−6.83 ± 0.66
	**NM-212**	−11.3 ± 0.1	−11.7 ± 0.4
RPMI	**NM-200**	−10.7 ± 0.5	−16.7 ± 2.0
	**NM-203**	−11.2 ± 0.3	−21.4 ± 1.4
	**NM-100**	−11.4 ± 0.7	−0.154 ± 1.830
	**NM-101**	−11.8 ± 0.2	−0.189 ± 1.740
	**NM-212**	−11.2 ± 0.2	−11.7 ± 0.8

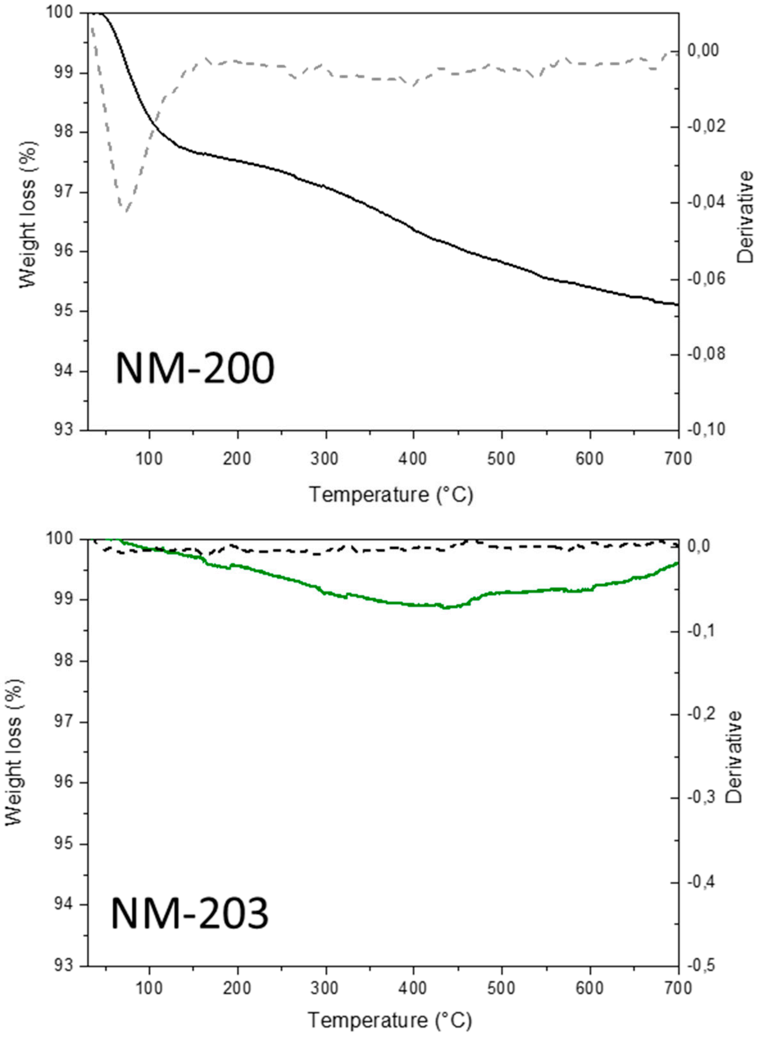

Figure S1. TGA weight loss (full line) and derivative (dotted line) curves for NM-200 and NM-203, under N_2 flow (TGA heating ramp = 15 °C/min, N_2 flow rate = 35 mL/min).

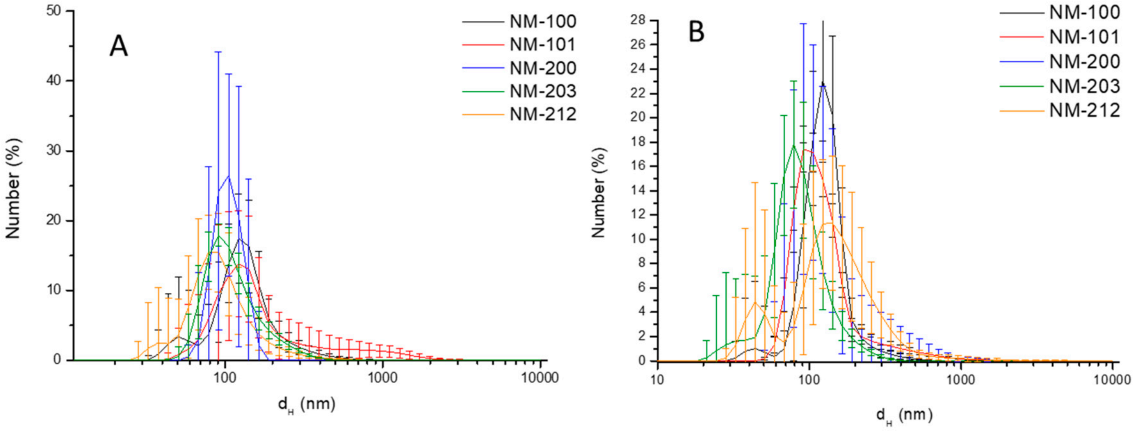

Figure S2. d_H distribution (number)of stock suspensions. (**A**) traditional protocol (**B**) standardized protocol.

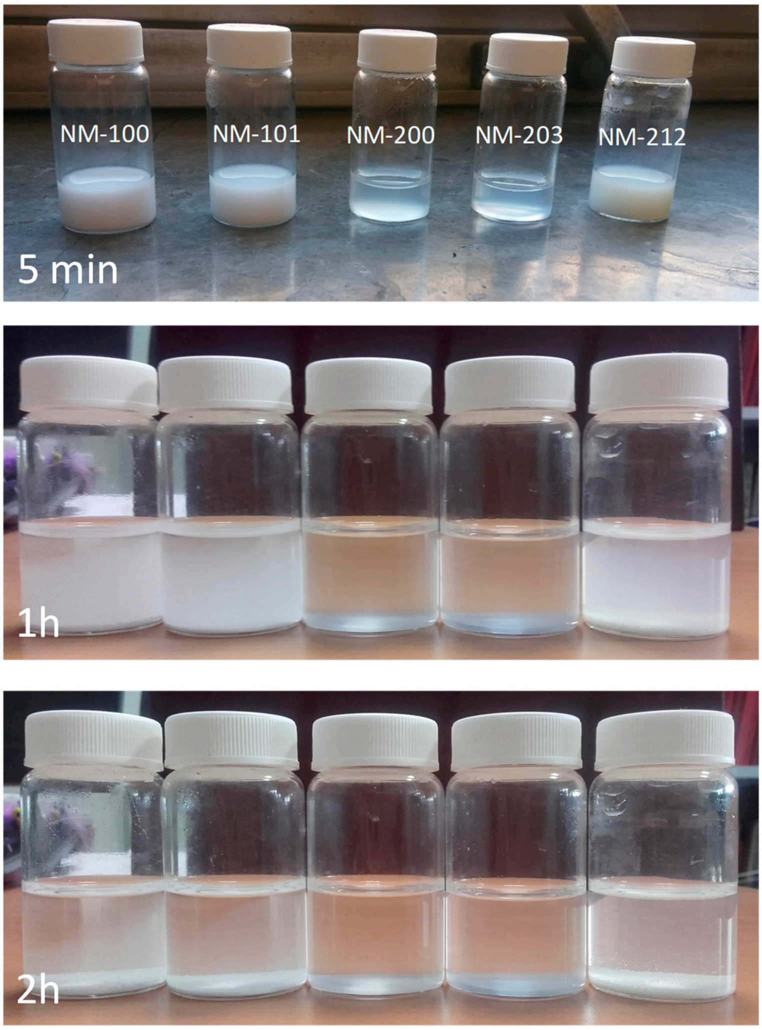

Figure S3. Images of the stock suspension prepared by the traditional protocol just after sonication, after 1 h and after 2 h.

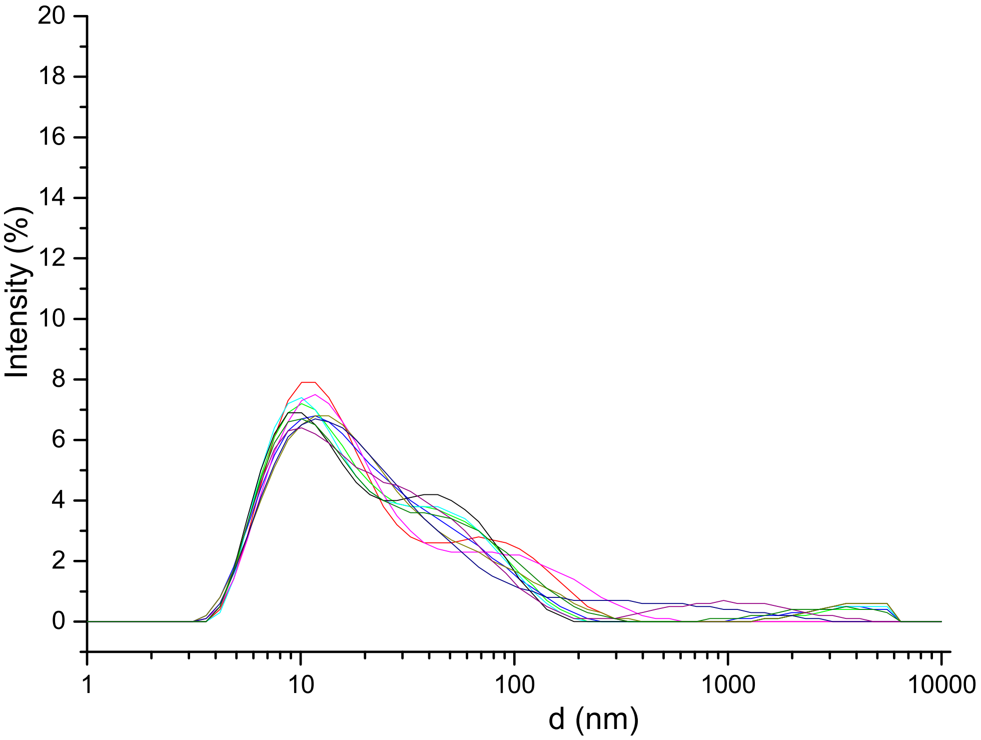

Figure S4. DLS analysis of the RPMI media.

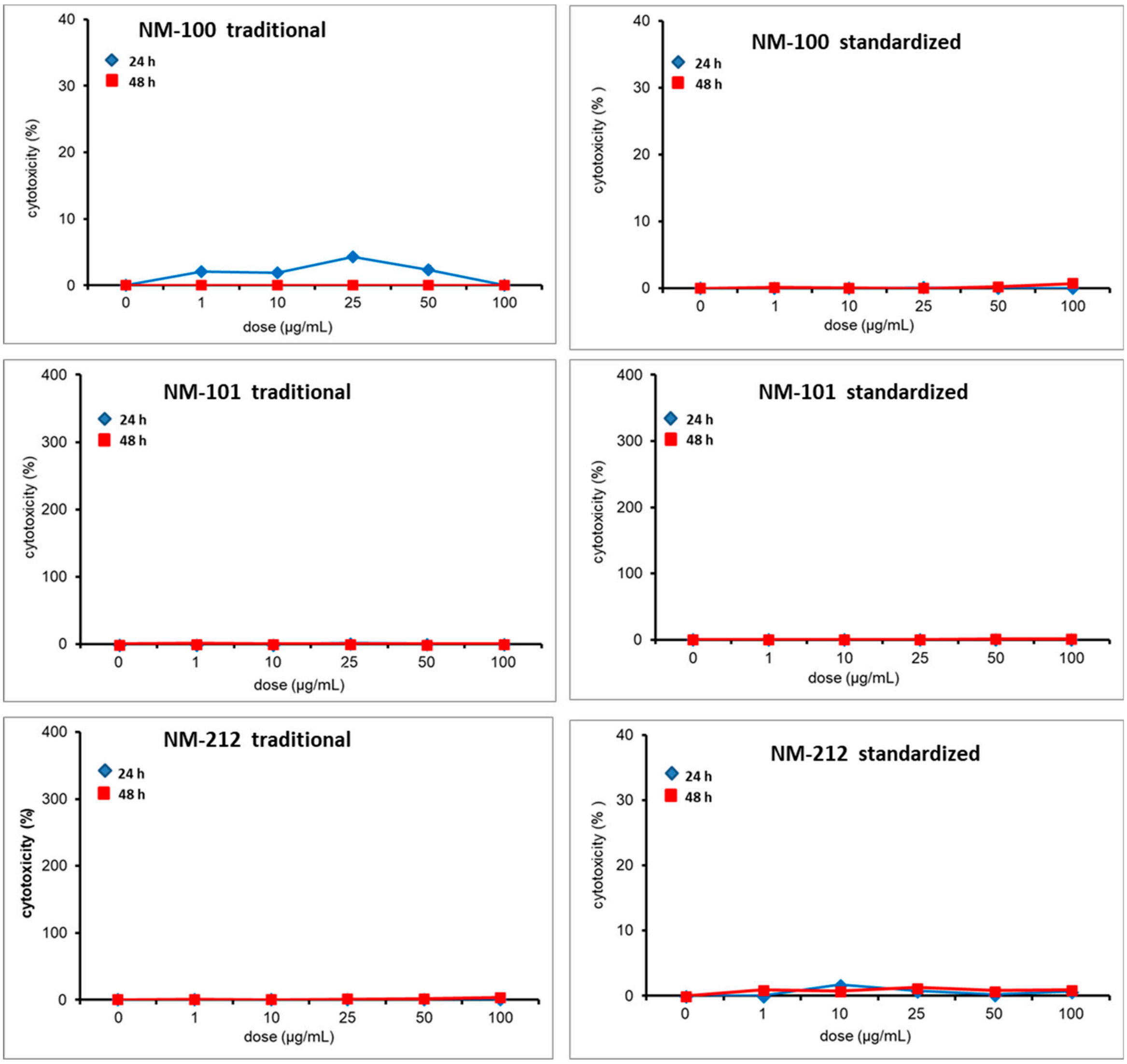

Figure S5. Effect of the dispersion protocols on the cytotoxicity of NM-100, 101 and 212 toward THP-1 cells measured as LDH leakage.

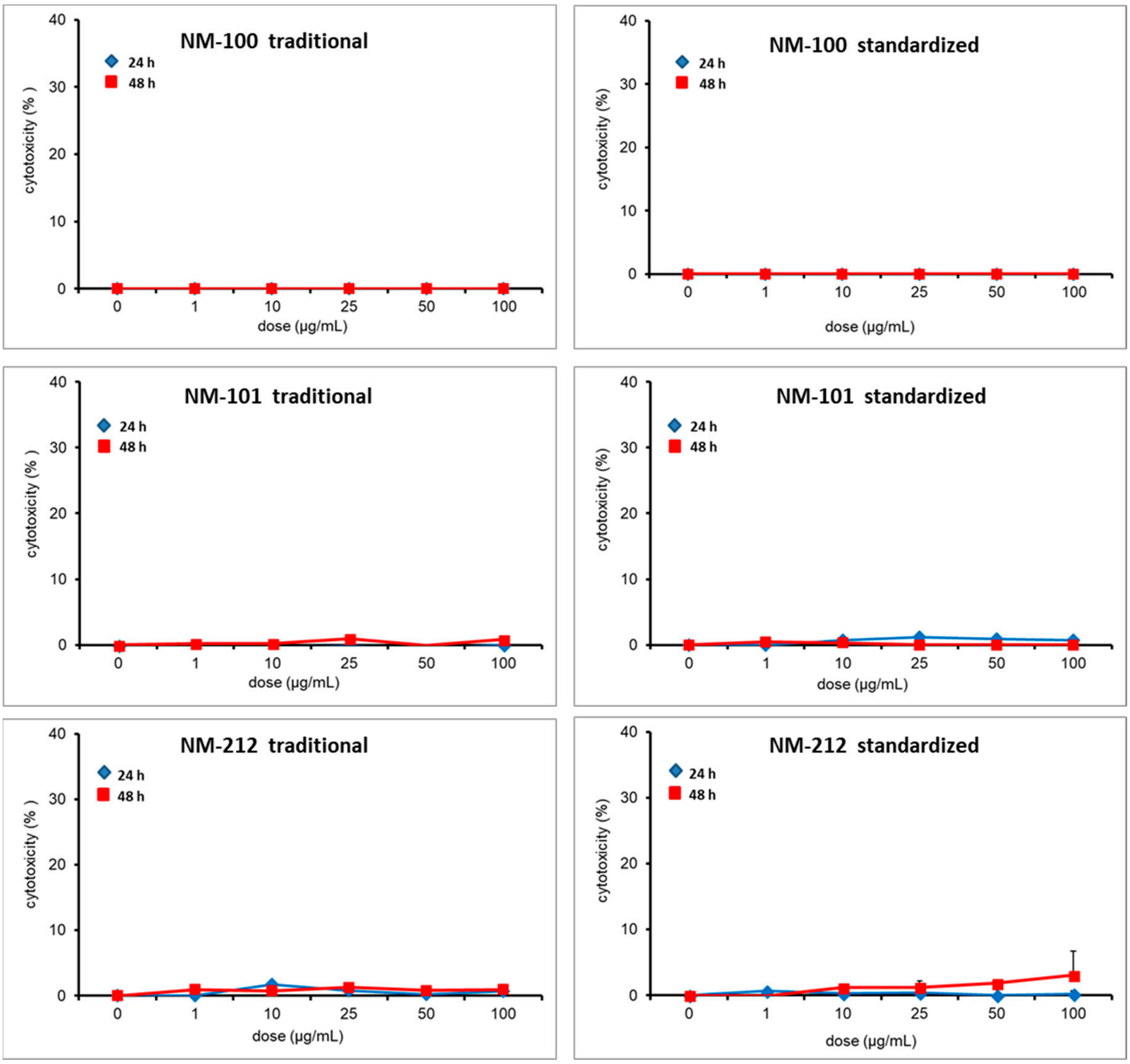

Figure S6. Effect of the dispersion protocols on the cytotoxicity of NM-100, 101 and 212 toward RAW 264.7 cells murine macrophages measured as LDH leakage.

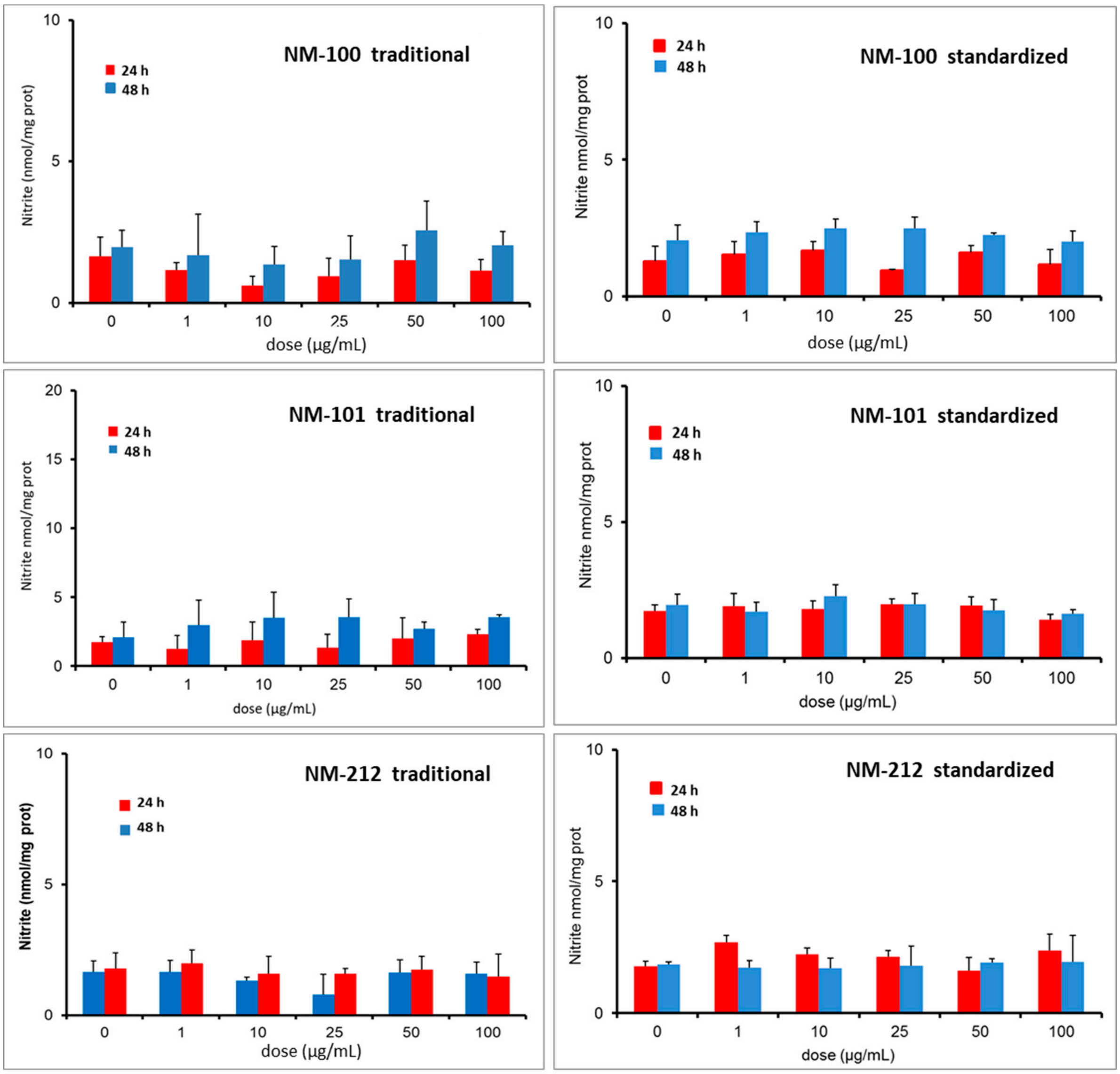

Figure S7. Effect of the dispersion protocols on the induction of NO release by RAW 264.7 murine macrophages by NM-100, 101 and 212.
